# Towards understanding the roles of position and geometry on cell fate decisions during preimplantation development

**DOI:** 10.1016/j.semcdb.2015.09.006

**Published:** 2015-12

**Authors:** John S. Biggins, Christophe Royer, Tomoko Watanabe, Shankar Srinivas

**Affiliations:** a Cavendish Laboratory, University of Cambridge, Cambridge, UK; b Department of Physiology Anatomy and Genetics, University of Oxford, Oxford, UK

**Keywords:** Preimplantation mouse embryos, Cell lineage, Hippo pathway, Cell geometry, Mechanical forces, Computational modelling

## Abstract

The first lineage segregation event in mouse embryos produces two separate cell populations: inner cell mass and trophectoderm. This is understood to be brought about by cells sensing their position within the embryo and differentiating accordingly. The cellular and molecular underpinnings of this process remain under investigation and have variously been considered to be completely stochastic or alternately, subject to some predisposition set up at fertilisation or before. Here, we consider these views in light of recent publications, discuss the possible role of cell geometry and mechanical forces in this process and describe how modelling could contribute in addressing this issue.

## 1 Introduction: environmental influences and stochasticity in lineage segregation

The first lineage segregation event during mouse embryogenesis is the formation of the pluripotent inner cell mass (ICM) and trophectoderm (TE) at a stage when the embryo is composed of approximately 32 cells. The TE arises from cells on the outside of the embryo while the ICM arises from those inside cells enclosed by the TE. Subsequent to this, the ICM further differentiates into the pluripotent epiblast and overlying primitive endoderm. Concomitantly, the blastocyst undergoes morphogenetic changes, as the cavity expands. The molecular genetic basis for the differentiation of these early cell types has been extensively studied and several excellent reviews of the field exist [1], [2], [3].

However, in the past few years there have been exciting findings that have started to reveal the mechanisms by which blastomeres can incorporate information about their physical environment into the ‘internal’ genetic imperatives that drive their differentiation. In this perspective piece, we look at the potential role of geometry and mechanics on cell fate determination in the early embryo. We use *geometry* to refer to the relative positions of cells within the embryo particularly with respect to each other and *mechanics* to refer to the forces on cells, irrespective of position. We discuss ideas relating to how the actin cytoskeleton, apical polarity complex proteins and the YAP transcriptional regulator provide mechanisms by which blastomeres can incorporate cues arising from geometry or their mechanical environment into the decision making process determining their fate. We consider ways in which computational approaches can help us understand how these varied inputs can act together to give consistent lineage allocation outcomes.

The role of environmental influences on lineage segregation is closely linked to the question of whether some sort of lineage information is present in the embryo as early as at the time of fertilisation – whether the course of development is in some way already *determined* in the way, for example, axial information is pre-determined in the *Drosophila* embryo or, as an extreme example, cell fate is determined in *Caenorhabditis elegans* [4], [5], [6]. In these organisms, lineage segregation is in a sense ‘hardwired’ into the zygote at fertilisation by the localisation of specific molecular determinants and there is little need (and possibly room) for environmental influences on the course of development. Given that the highly regulative nature of mouse development is beyond debate, in the murine context such pre-determination is described rather in terms of a predisposition, in the unperturbed state, of specific cells to particular fates [7].

The opposing view is that lineage determination is a *stochastic* process [8], [9]. Since this term is subject to interpretation, we note that we use stochastic to mean that the outcome of a particular process cannot be predicted with certainty given the starting conditions, though one can ascribe a probability to particular outcomes. In this view environmental input such as the position of a blastomere or the forces it is subject to could play a significant role in lineage determination. They could act as a source of stochasticity, for example through the ‘chance’ position of a blastomere affecting its fate. Equally, they could act conversely as a regulatory buffer against underlying sources of stochasticity such as transcriptional noise.

Geometry as a regulatory buffer sounds abstract, but is made concrete by a simple example. If you break a strand of dry spaghetti by holding the two ends and bending, it infamously typically breaks in two places along its length [10] giving three pieces which can be thought of as two ‘outside’ pieces and one ‘inside’ piece. Despite considerable stochasticity in the precise places the spaghetti breaks, in cell-fate terms the output appears deterministic (one inside and two outside pieces every time) because of the starting geometry.

In practice, the modes of embryonic development in different species fall on a spectrum. At one end lies predetermined development, where an embryonic template is laid down at or shortly after fertilisation and every healthy embryo produces the same lineage tree. At the other end lies highly stochastic development where, at early stages, there is no template and cells behave independently, leading different embryos to produce completely different lineage trees. Stochastic development *requires* regulation so that the differing lineage trees produce consistent embryos. Mammalian embryos are certainly not completely predetermined, but there is uncertainty about where along this spectrum they lie. Advocates of “predisposition” suggest that cell fates are biased by inherited characteristics such as the sperm entry point, leading to a template that results in somewhat homogenous lineage trees [11]. Advocates of stochasticity typically emphasise the role of external perturbations and chance variations in cell positions, division angles, *etc.* in driving divergences between lineage trees [9]. However, the two positions are neither polar opposites nor irreconcilable, rather the question is one of degree: what elements of stochasticity and predisposition explain the observed variation between mammalian embryos? In this context, computational modelling can make an important contribution to understanding the possible influence of geometry and mechanics on lineage segregation and the extent to which decisions made in the early embryo are stochastic, whether constrained by geometry or not.

## 2 Modelling stochasticity in early embryonic development

At its best theory does not just describe, it unifies, explains and predicts. By this yardstick quantitative modelling of mammalian embryos is itself embryonic, not yet even describing how and when early cell fates are specified (or even a clear sense of what fate specification means) let alone elucidating unifying design principles or providing insights into developmental trade-offs. The difficulty has several origins, including that mammalian embryos appear to sit uncomfortably somewhere between determinism and stochasticity, and that they have rather too many cells to elegantly model each individually but too few to model as a continuum.

However, we see several avenues for progress. Firstly, we take inspiration from recent advances in the stochastic modelling of stem-cell maintained tissue [12], [13], [14], [15]. The key to much of this success has been modelling stem-cell division and fate choices as independent random processes, a surprisingly simple assumption which allows the modelling to be done in the language of random-branching processes. In maintenance problems this leads to nice mathematical results because maintenance is quasi-static, so the branching processes are “critical”. These results are surely lacking in embryos, where cell number grows exponentially, but we expect similar stochastic modelling of cell division and fate choices will nonetheless be useful [16].

The need for stochastic models is illustrated by our previous work [17]. At the 32 cell stage, mouse embryos reliably produce 11–12 ICM cells and 20–21 trophectoderm cells, but we saw substantial variation in the lineage trees that give rise to these consistent results. For example, we saw embryos where all 16-cell-stage blastomeres gave rise to daughters with matching fates, and examples where five 16-cell-stage blastomeres had one ICM daughter and one trophectoderm daughter. These consistent results from varying pathways are indicative of the balance between stochasticity and regulation that a successful model must capture. Our data sets were not large enough to meaningfully test stochastic models. For this reason, we look forward to full linage trees for much larger numbers of embryos in the future.

A good place to start is likely to be with cell division angles at the 8- to 16-cell transition. In our recent work we showed that the division angle chosen by cells at this point is not isotropically distributed, but biased towards asymmetric divisions. A simple stochastic model, inspired by the aforementioned stem cell maintenance, is that each cell “draws” a division angle from this biased distribution independently. This independence is, with a large enough data set, a statistically testable prediction, as it demands that the levels of variation within an embryo and between embryos must be consistent. If true, this isolates a significant source of stochasticity and we should next investigate how it is regulated, asking to what extent is the resultant inter-embryo variation still present before the next division round. If it is not true, we learn that cell choices of division angle are coordinated, indicating the 8-cell embryo is to some extent patterned.

**Fig. 1:**
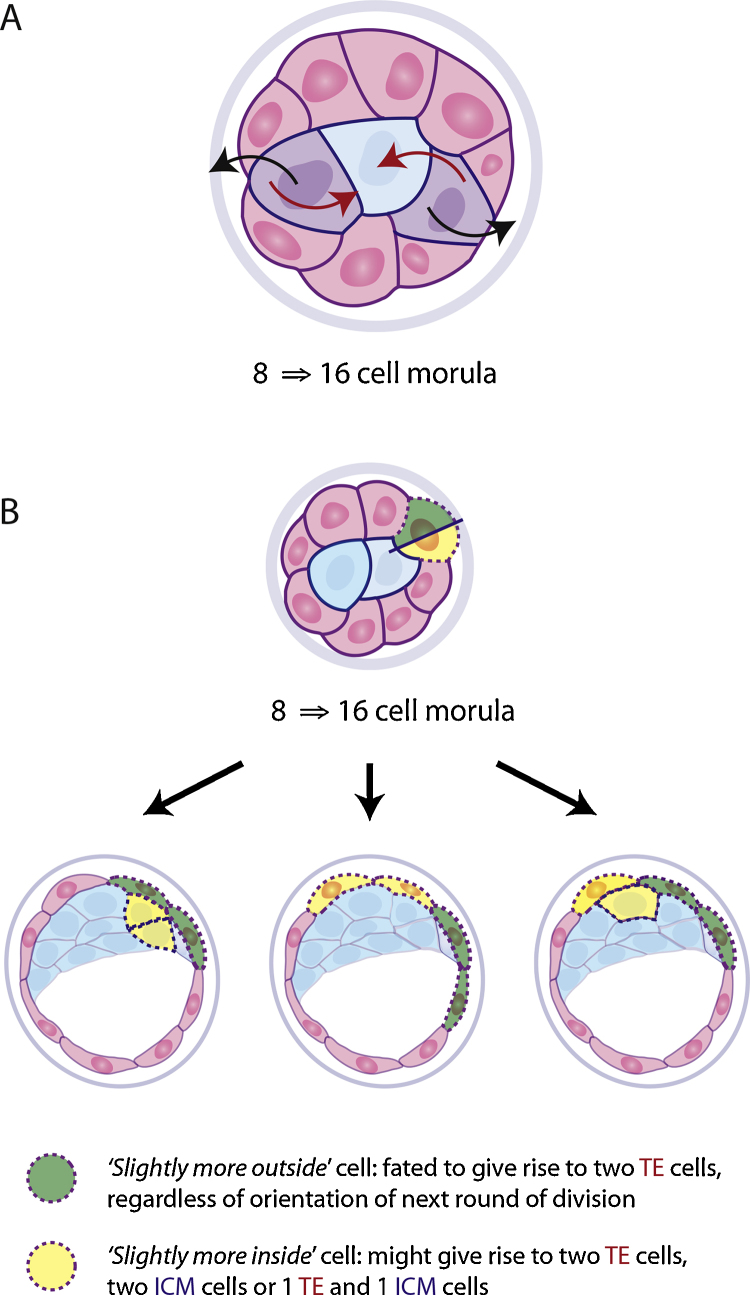
Movement and fate of cells in the morula. (A) Between the 8- and 16-cell stages, blastomeres can move inside or outside. Red arrows indicate cell movement towards the inside of the embryo and black arrows indicate cell movement towards outside of the embryo. (B) During this stage, the daughter cells show a spectrum of membrane exposure to the outside. Only the ‘slightly more inside’ daughter of an almost symmetric division can give rise to any ICM cells. Slightly more outside cells from such divisions are limited to the TE fate.

Going beyond the 8- to 16-cell division, we run into the added difficulty of geometry which can, in fact, provide a degree of regulation. A random-branching stochastic model of cell fate choice would yield a non-zero chance of all cells ending up on the inside or on the outside, both of which are clearly geometrically impossible. A very pertinent question is whether geometry can provide enough regulation on its own to explain early term embryo lineage trees. A good framework to start testing this idea in would be a stochastic analogue of the traditional inside-outside model [18] in which cells divide stochastically and independently and the position of the resultant daughter cells determines their ultimate fate. In such a model geometry will indeed provide a degree of regulation, making the final numbers of ICM and trophectoderm cells less variable than a “well-mixed” geometry free stochastic model (Fig. 1).

## 3 The Hippo pathway – how cells sense geometry

Until relatively recently it was unclear how a blastomere could ‘sense’ its position in the embryo. Recent studies have brought a refreshing new view on how TE and ICM are generated, revealing that the Hippo pathway and its effectors YAP and TAZ (YAP/TAZ) are central to this process [19], [20], [21] and provide a mechanism for not only sensing but also responding to the position of blastomeres within the embryo.

The Hippo pathway is a signalling cascade whose core components are broadly conserved from flies to mammals and acts in many different cellular contexts [22], [23]. It results in the phosphorylation of the transcription cofactors YAP/TAZ by Lats kinases. Phosphorylated YAP/TAZ are either retained in the cytoplasm through their interaction with 14-3-3 or degraded *via* proteasome-targeted degradation. In the dephosphorylated state, YAP/TAZ can enter the nucleus and act as co-factors for a variety of transcription factors in the nucleus such as TEAD4, thereby influencing their activity [24].

The Hippo pathway plays a major role in the early mouse embryo, as recently reviewed in detail [25], [26]. Briefly, a combination of cell adhesion and apical–basolateral polarity converts positional information into cell fate decisions by differentially regulating the Hippo pathway. In inside cells, cell–cell contacts turn the Hippo pathway on, leading to the phosphorylation and inactivation of YAP/TAZ. As a result, YAP/TAZ are unable to enter the nucleus to interact with TEAD4, which remains inactive as it does not have a transactivation domain. This allows pluripotency markers to flourish and establish ICM fate [21].

**Fig. 2:**
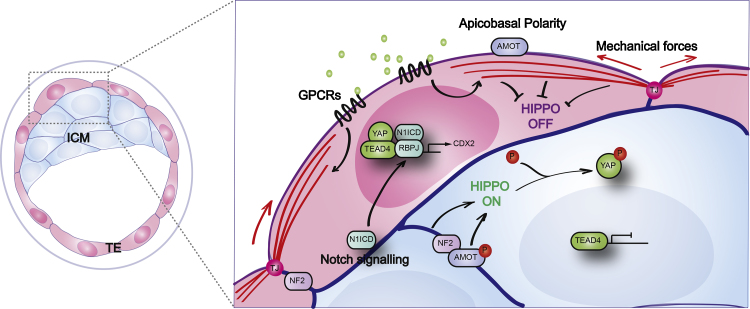
Position-dependent regulation of cell fate in the preimplantation embryo: known and hypothetical mechanisms. Regulation of the Hippo pathway is central to the first cell fate decision. In inside cells, a junctional complex comprised of NF2 and AMOT phosphorylated at S176 activates the Hippo pathway. This leads in turn to the phosphorylation of YAP and TAZ and their exclusion from nuclei, rendering them transcriptionally inactive. In outside cells, apicobasal polarity leads to the sequestration of AMOT dephosphorylated at S176 to the apical domain, resulting in the inactivation of the Hippo pathway. Unphosphorylated YAP is therefore able to go to the nucleus where it can bind to TEAD4 to activate CDX2 transcription and drive TE fate. Other factors, such as mechanical forces, GPCR and PCP signalling may act as cell shape and position sensors and lead to the activation of YAP and TAZ in outside cells principally *via* modulation of the actin cytoskeleton. Notch signalling, activated in outside cells only *via* an unknown mechanism, contributes to the transcription of *Cdx2* to establish TE fate. TJ: tight junctions. Actin filaments are represented in red. The apical domain of TE cells is outlined in purple and cell–cell junctions are delineated in blue.

Apical–basolateral polarity is required for the formation of the TE and inhibition of key polarity components such as Par3 and aPKC in blastomeres causes them to favour contributing to the ICM [27]. Experiments inactivating the aPKC/Par6 polarity complex in outer cells demonstrate that apical–basolateral polarity is required to inhibit the Hippo pathway by segregating AMOT, dephosphorylated at serine 176, to the apical membrane. When AMOT is localised to the apical membrane, it is unable to interact with NF2 at cell–cell junctions, preventing Hippo activation [19], [28]. This results in the relocalisation of YAP/TAZ to the nucleus, and the TEAD4-dependent transcription of TE specific genes such as *Cdx2* [19]. Recent findings suggest that Notch signalling also plays an important role in directly promoting the transcription of *Cdx2* in outside cells in collaboration with YAP/TEAD4 [29]. This implies that, in addition to apical–basolateral polarity, other mechanisms are in place to define cell position and determine cell fate within the preimplantation embryo (Fig. 2). However, it remains unclear how Notch signalling is activated in TE cells and future studies will establish the role of ICM cells for instance in this mechanism.

These data show that YAP/TAZ nuclear localisation (and therefore *Cdx2* expression and TE formation) is dependent on proper establishment of apical–basolateral polarity. As only outside cells are polarised and have such a complex, this provides a way by which a blastomere can ‘sense’ its position in the embryo, based on the existence (or not) of an apical polarity complex within the cell.

## 4 Actin as an integrator of mechanical cues influencing lineage segregation and morphogenesis

In addition to geometry, embryos are also constrained by physics. It is possible that outside cells experience a different range of forces than the inside cells they are stretched over. Although the establishment of apical–basolateral polarity in outside blastomeres is now accepted to be a crucial step in regulating YAP/TAZ in the context of the first cell fate decision, it remains relatively unexplored whether mechanical cues also participate in their regulation. The establishment of polarity is accompanied by changes in the organisation of the actin cytoskeleton which may, as a consequence, relay information on cell shape and mechanical forces applied by the microenvironment. Interestingly, mechanical signals exerted by cell shape and extra-cellular matrix stiffness can be sensed by YAP and TAZ *via* the actin cytoskeleton in the context of *in vitro* cell cultures [30], [31], [32], therefore raising the question of whether YAP/TAZ may also play a role in mechanotransduction in the preimplantation embryo (Fig. 2).

In recent years, great strides have been made in understanding how actin dynamics and architecture can influence the hippo pathway *in vitro* in cell culture and *in vivo*, mainly using *Drosophila* as a model system (reviewed in [33]). However, it remains unclear which subcellular actin structures are directly involved in the mechanosensory properties of YAP/TAZ. This question is relevant in the context of preimplantation embryo development as different actin structures are progressively established. Most notably, at compaction when blastomeres establish an apicobasal polarity axis, actin is recruited apically following the phosphorylation of Ezrin by aPKC [34]. Considering the physical nature of compaction and the importance of the forces generated by the actomyosin cortex during this process [35], [36], together with the notable increase in nuclear YAP from the 2- to 8-cell stage [21], it will be important to establish whether a link exists between cortical tensions and the Hippo pathway in this context. For now, it remains unclear how these F-actin-containing rings or cap structures at the apical membrane may be involved in mechanosensing and, instead, they may help define whether daughter cells should adopt a symmetric or asymmetric fate [37].

Other F-actin interacting structures may be involved in mechanosensing during preimplantation development. For instance, F-actin is highly associated with tight junctions *via* ZO-1 [38]. During preimplantation development, the formation of tight junctions is a multi-step process which starts at the 8-cell stage and continues until the blastocyst stage in outside cells only [39]. Moreover, it has been shown that ZO-1 is involved in morula to blastocyst transformation [40]. Tight junction maturation therefore coincides with the establishment of the TE lineage and blastocoel cavity formation, suggesting that F-actin structures present at TJ may be involved in sensing local tensions to influence these processes. This is supported by findings directly linking tight junction associated proteins such as ZO-1 and ZO-2 to the regulation of YAP and TAZ [41], [42].

Furthermore, underlining the complexity of YAP/TAZ regulation, a number of upstream signalling pathways, such as GPCR signalling have been shown to affect their activity [24], [43], [44], [45]. It is conceivable that the actin cytoskeleton acts as the main mediator of these signals (Fig. 2). Recent studies have further highlighted this level of complexity, by unravelling the YAP/TAZ protein interaction network in cell lines using interaction proteomics [46], [47], [48]. Applying this type of approach to the preimplantation embryo may prove to be challenging, mainly due to the scarcity of material obtained from a single preimplantation embryo. However, as technological efforts are made to reduce the number of cells required for these types of approaches, it may become possible for instance to establish the protein interaction network of YAP/TAZ in outside *versus* inside cells or identify specific transcription programs activated by YAP/TAZ in outside cells which would ultimately lead to the identification of new TE specific markers.

Beyond mechanosensing, mechanical forces also directly distort and sculpt cells, and can thus drive the formation of complex morphologies directly rather than simply being sensed and triggering internal developmental pathways. Recently several examples of mechanical forces and elasticity (rather than any underlying chemical or biological patterning) driving the emergence of complex shapes during morphogenesis have come to light [49], [50], [51], [52], [53]. In each of these cases, the morphogenesis is underpinned by a physical instability that spontaneously generates a radical and complex shape change.

Early mouse embryos undergo a radical shape change when they undergo blastulation. We suggest that blastulation is also underpinned by a physical instability, namely solid-cavitation: a small pressurised cavity in a solid medium will at a critical pressure (2.5 times greater than the shear modulus) inflate to a macroscopic size [54], [55]. In the embryo context, the pressure is surely osmotic pressure generated by ion pumps [56], but it is unclear whether the resistive forces are elastic, viscous or surface tension or indeed important at all. A few previous studies have shown that the blastocoel fluid has an excess ionic concentration of around 5 mM [57], enough to generate 12 kPa of pressure, and hence cavitate a solid with a shear modulus of 5 kPa, eerily close to the estimated elastic modulus of mammalian embryos [58]. To test this, accurate measurements of the osmotic pressure [57], [59], elastic modulus [58], [60] and surface tension [61] will need to be taken, to verify its plausibility. Ultimately a destructive experiment is required: the cavity must be pierced, to investigate whether equalising the pressures causes it to elastically deflate. If correct, this model might imply that the large shape changes of cells during blastulation are mechanically driven, and cause specialisation rather than being caused by it. Secondly, it would imply that the position of the cavity is determined simply by the location of the first micro-cavity to achieve cavitation pressure, not by pre-pattering of the morula.

Testing the importance of mechanosensing and the specific magnitude of forces in the preimplantation embryo is not trivial and the tools are not readily available. However, FRET (Förster resonance energy transfer) based approaches have been applied successfully *in vitro*, in cell culture experiments [62], [63], [64]. These methods allow the measurement of forces across specific proteins with extreme sensitivity at subcellular level. In the case of vinculin for instance, a tension sensor module was inserted between its head and tail domains which interact with talin at focal adhesions and the actin cytoskeleton respectively. This tension sensor module is composed of an elastic domain inserted between two fluorophores that undergo efficient FRET. Under tension, the distance between the two fluorophore increases, therefore affecting the efficiency of FRET [63]. It may be possible to use similar approaches in the embryo to measure the forces experienced by actin for instance, ideally using genetically modified mouse lines and *in vitro* development of embryos.

## 5 Integrative approaches to early embryonic development

Simple cellular-resolution vector representation of developing blastocysts can be a powerful way to capture basic quantitative information about changing physical characteristics of component blastomeres such as surface area, volume and orientation of division [17], [65]. Such models also provide a useful scaffold on which to build other relevant data about the developing embryo. With advances in live imaging techniques, the time-lapse recordings that form the basis for these vector models can be captured at higher resolution (both temporal and spatial) as well as over increased durations, to cover development from fertilisation to implantation stages. Combining the FRET reporters of force described above or reporters of the actin cytoskeleton such as LifeAct [66] one can potentially start to map forces experienced by cells and the state of the cytoskeletal architecture onto the vector representations of their development. With rapid advances in single-cell sequencing technologies [67], [68], one could also envisage sequencing the individual cells of such imaged embryos, to capture information about their transcriptional and epigenetic states and integrate this into the context of their recorded cellular history. We, however, need to develop powerful new modelling approaches to build these diverse data types into meaningful theoretical models that can provide insights into normal development.

Bringing together stochasticity, geometry, physics and chemical regulation in a single analytic model is probably beyond hopeless, but doing so on a computer is surely not. Indeed, the few cells in early embryos make them a promising candidate for cell-by-cell simulations [69], [70], [71] in which each cell is described both by its shape and position in space and by internal state variables. Such simulations necessarily include geometric effects, and there is no reason they should not include full and realistic mechanical effects, such as adhesion, osmotic pressure and elastic forces in addition to information about molecular interactions, offering a natural testing ground for the interplay between regulation and stochasticity that helps give rise to the early embryo.
